# Health systems performance for hypertension control using a cascade of care approach in South Africa, 2011–2017

**DOI:** 10.1371/journal.pgph.0002055

**Published:** 2023-09-07

**Authors:** Mariet Benade, Zandile Mchiza, Rafeya V. Raquib, Sridevi K. Prasad, Lily D. Yan, Alana T. Brennan, Justine Davies, Nikkil Sudharsanan, Jennifer Manne-Goehler, Matthew P. Fox, Jacob Bor, Sydney B. Rosen, Andrew C. Stokes

**Affiliations:** 1 Department of Global Health, Boston University School of Public Health, Boston, Massachusetts, United States of America; 2 Faculty of Health Sciences, Health Economics and Epidemiology Research Office, University of the Witwatersrand, Johannesburg, South Africa; 3 Non-Communicable Disease Research Unit, South African Medical Research Council, Cape Town, South Africa; 4 School of Public Health, University of the Western Cape, Bellville, South Africa; 5 Department of Medicine, Weill Cornell Medicine, New York, New York, United States of America; 6 Department of Epidemiology, Boston University School of Public Health, Boston, Massachusetts, United States of America; 7 Department of Global Health, University of Birmingham, Birmingham, United Kingdom; 8 Heidelberg Institute for Global Health, Heidelberg University, Heidelberg, Germany; 9 Division of Infectious Diseases, Massachusetts General Hospital, Boston, MA, United States of America; Institute of Public Health Bengaluru, INDIA

## Abstract

Hypertension is a major contributor to global morbidity and mortality. In South Africa, the government has employed a whole systems approach to address the growing burden of non-communicable diseases. We used a novel incident care cascade approach to measure changes in the South African health system’s ability to manage hypertension between 2011 and 2017. We used data from Waves 1–5 of the National Income Dynamics Study (NIDS) to estimate trends in the hypertension care cascade and unmet treatment need across four successive cohorts with incident hypertension. We used a negative binomial regression to identify factors that may predict higher rates of hypertension control, controlling for socio-demographic and healthcare factors. In 2011, 19.6% (95%CI 14.2, 26.2) of individuals with incident hypertension were diagnosed, 15.4% (95%CI 10.8, 21.4) were on treatment and 7.1% had controlled blood pressure. By 2017, the proportion of individuals with diagnosed incident hypertension had increased to 24.4% (95%CI 15.9, 35.4). Increases in treatment (23.3%, 95%CI 15.0, 34.3) and control (22.1%, 95%CI 14.1, 33.0) were also observed, translating to a decrease in unmet need for hypertension care from 92.9% in 2011 to 77.9% in 2017. Multivariable regression showed that participants with incident hypertension in 2017 were 3.01 (95%CI 1.77, 5.13) times more likely to have a controlled blood pressure compared to those in 2011. Our data show that while substantial improvements in the hypertension care cascade occurred between 2011 and 2017, a large burden of unmet need remains. The greatest losses in the incident hypertension care cascades came before diagnosis. Nevertheless, whole system programming will be needed to sufficiently address significant morbidity and mortality related to having an elevated blood pressure.

## Introduction

Hypertension is a major contributor to global morbidity and mortality, resulting in an estimated 10.4 million deaths and 218 million disability adjusted life years (DALYs) in 2017 [1]. Despite the availability of inexpensive evidence-based interventions such as lifestyle modification and pharmacotherapy, hypertension prevalence and complication rates have continued to rise globally [2–4]. This trend affects low- and middle-income countries (LMIC’s) disproportionately. More than a fifth (21%) of all deaths in LMIC’s are attributed to hypertension and prevalence has increased by 7.7% between 2000 and 2010, while it has decreased by 2.6% in high-income countries [5,6].

In South Africa, the prevalence of hypertension was 46% in women and 44% in men in 2016 [7]. Among people living with hypertension, the proportion with controlled hypertension was only 20% in women and 13% in men that year, suggesting that substantial gaps in coverage and quality of hypertension care remain [7]. Previous work has found that low rates of diagnosis and treatment among those with hypertension create a high burden of unmet need for hypertension management [7,8]. A cost-effectiveness analysis in 2019 concluded that increasing hypertension diagnosis and treatment in South Africa would substantially reduce DALYs attributed to the condition while also cutting net health care costs related to complications of hypertension, potentially saving the South African health care system $24, 902 per DALY averted [9].

Although the South African government has undertaken numerous health systems reforms in recent years with the aim of strengthening management of chronic non-communicable diseases, including improved guidelines and the *Strategic Plan for the Prevention and Control of Non-Communicable Diseases 2013–2017*, the extent to which new policies have translated to population-wide improvements in hypertension control remains unclear [10–12].

One approach to tracking the appropriateness of health systems interventions are care cascades. This approach can identify the specific stages of care at which interventions should be focused to improve chronic disease control, and monitor progress over time, as suggested by the 2018 Lancet Global Health Systems Strengthening Commission [13]. By measuring how individuals move through a health care system from screening and diagnosis to treatment and, ultimately, control, care cascades identify potential gaps in patient management across the care continuum and capture which components of health service delivery require the most improvement [14,15].

Despite their potential value, current care cascade analyses for hypertension are generally based on prevalent samples assessed at a single point in time, and thus cannot capture time trends or distinguish between historical and current health system weaknesses [16]. More nuanced health systems planning requires repeated assessment of cohorts with new onset of disease to allow inferences of trends in care cascades to attributes of the contemporary health care system, as has recently been advocated for in HIV cascades [17].

In the present study, we evaluate changes in the cascade of care for hypertension in South Africa over the period 2011–2017 using data from the National Income Dynamics Study (NIDS). We develop a novel longitudinal approach to analyzing hypertension care cascades in which we examine successive cohorts of people who newly develop hypertension over the defined period. This incident cascade approach offers a clearer depiction of health system performance at specific points in time, as it allows for a consideration of movement of new cases through the cascade in a specified time period. Using data from all five waves of NIDS, we use incident cascades to assess current health system performance and the potential effects of policies introduced over the study period [18–22].

## Methods

### Design and setting

This study uses data from the first through the fifth waves of the National Income Dynamics Study (NIDS) [18–22]. NIDS is a nationally representative panel survey that was implemented by the South African Labour and Development Research Unit at the University of Cape Town’s School of Economics to track changes in the structure and living conditions of South African households and the living conditions and the well-being of individual household members over time.

The first wave of data was collected in 2008. The initial sample population consisted of 28,000 individuals from 7,300 households across South Africa. Using a stratified, two-stage cluster sample design, households were first stratified using 53 district councils with 400 primary sampling units, then randomly selected within each stratum [23]. Four subsequent waves collected data from the same households and their individual members in 2011, 2013, 2015 and 2017. The sample sizes differed slightly for each wave due to attrition and individuals moving in and out of households [23]. Attrition rates were highest among White and Indian/Asian participants and participants in high income categories [23].

Data were collected by trained interviewers during face-to-face interviews conducted in the participant’s preferred language. Similar questions were asked of participants at each wave of the survey. Questions relevant to this study included age, known illnesses (asked in free form), education level, and ethnicity (asked with a list of options). Blood pressure measurements were conducted at the end of each interview, with two measurements taken at five-minute intervals.

### Participants

We incorporated data on participants with at least two consecutive waves of complete data on relevant covariates, including hypertension diagnosis and treatment status and blood pressure measurements. We included individuals aged 35–79 at each wave (S1 Fig).

### Incident cohort design

To create our incident cohorts, we identified people who met our incident hypertension case definition at each wave of the NIDS study. We defined an individual as an incident case if they had high blood pressure (defined below) in wave two, three, four or five, and had not reported hypertension or had an elevated blood pressure in the previous wave. Using the newly hypertensive cases in each wave, we created incident cohorts of new cases of hypertension across all 4 waves (S2 Fig).

### Care cascade

**Fig 1:**
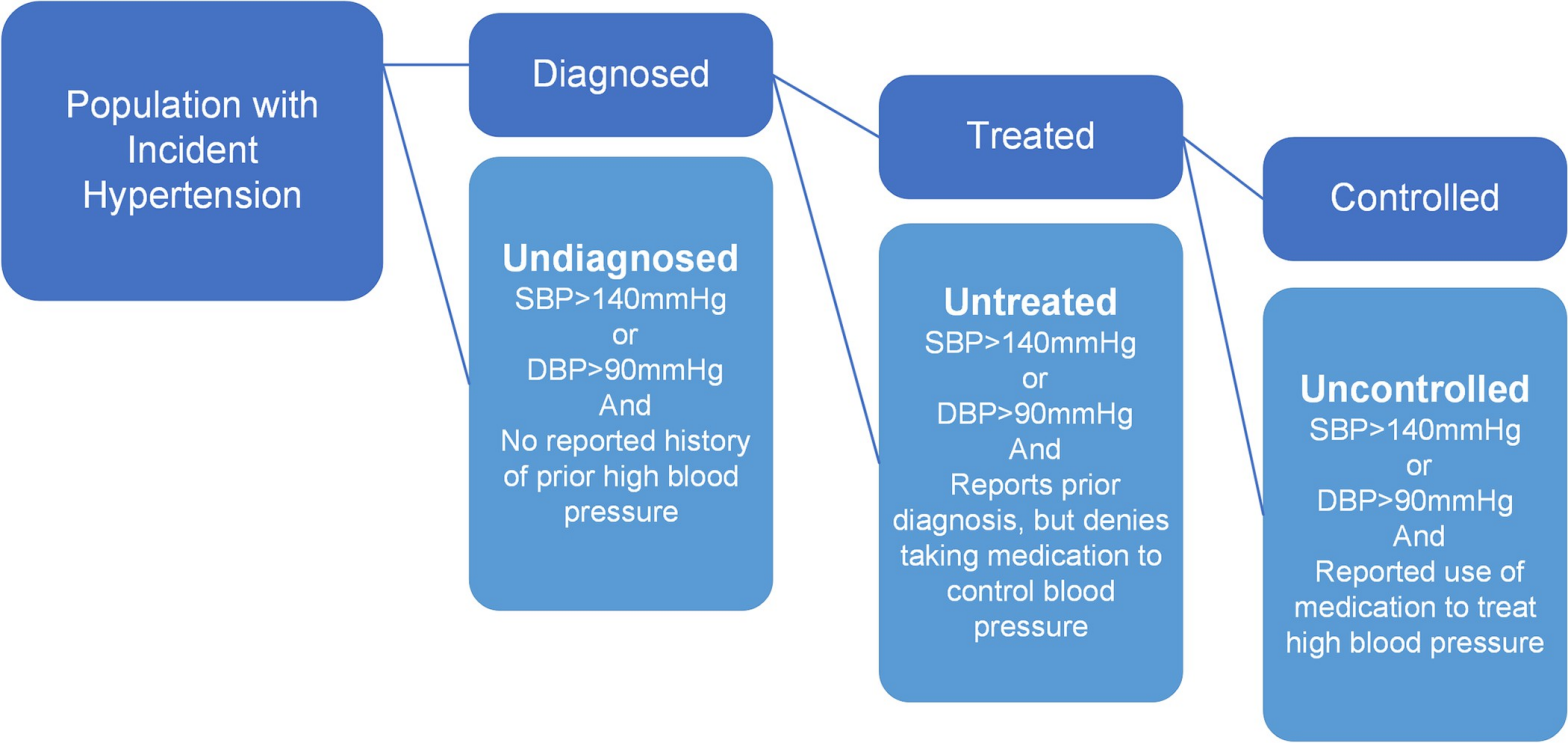
Conceptual design figure. SBP, Systolic Blood Pressure. DBP, Diastolic Blood Pressure.

We constructed hypertension care cascades for each incident cohort, which enabled us to calculate losses across the care continuum and the overall burden of unmet need for hypertension care. We defined a care cascade for hypertension with four stages: (1) hypertensive, (2) diagnosed, (3) treated and (4) controlled. Individual participants’ entry into each subsequent stage of the cascade was contingent on them having reached the previous stage (Fig 1).

Using the current South African guidelines, we defined an individual as hypertensive (stage 1) if their average systolic blood pressure of two measurements was equal or higher than 140 mmHg, their average diastolic blood pressure equal or higher than 90mmHg, or they self-reported taking anti-hypertensive treatment [24]. We defined participants as diagnosed (stage 2) if they answered “yes” to the question “Have you ever been told by a doctor, nurse or health care professional that you have high blood pressure?”.

Participants were determined to be “treated” (stage 3) if they reported being diagnosed and that they were taking medication for high blood pressure and “untreated” if they were not. Finally, participants were determined to be “controlled” (stage 4) if they had been diagnosed, were on treatment and had a measured blood pressure less than 140 mmHg systolic and 90 mmHg diastolic taken at the time of survey interview. Participants who answered “yes” to the treatment question but had a blood pressure greater than 140 mmHg systolic or 90 mmHg diastolic were determined to be “uncontrolled”.

We defined “unmet need” as the sum of the proportion of the sample who remained undiagnosed, untreated or uncontrolled.

### Covariates

We included variables on sex, age group, race, education, province, urban or rural residence, medical aid (a form of private health insurance), year of survey, income, known comorbidities, and blood pressure measurements at the previous wave. Age groups were 35–44 years, 45–59 years, and 60–79 years. Race was classified according to the official groupings used by the South Africa Bureau of Statistics [25]. For education we created a dichotomous variable that distinguished between those who completed schooling up to grade nine, which is compulsory in South Africa, and those who completed schooling beyond the ninth grade. Year of survey was derived from the survey wave in which the participant met the criteria of an incident hypertension case. We created a new ordinal variable that assigned participants to an income quartile based on their total household income. We considered patients to have a known comorbidity if they responded “yes” to if they had ever been told by a health care worker that they had diabetes, stroke, heart disease, or cancer.

### Statistical analyses

Hypertension estimates were age-standardized to the population of South Africa using the 2017 census report [25]. We calculated incidence rate ratios using a multivariable negative binomial regression across all waves on the outcomes of being diagnosed, treated or controlled. The main variable of interest was survey wave, as this allowed us to assess changes over time. The model was adjusted for the covariates described above. All regressions were implemented on the full sample of participants with incident hypertension.We generated predicted probabilities using marginal standardization while controlling for the same covariates. Our primary analysis was unweighted. To account for the survey design, each incident cohort was weighted by that wave’s sample weights [23]. All analyses were performed in Stata 16 (StataCorp, College Station, TX).

### Ethical considerations

Ethics approval for NIDS data collection for Waves 1 to 5 were granted by University of Cape Town’s Commerce Faculty Ethics in Research Committee. For Wave 5 additional ethics approval was granted by University of Cape Town’s Faculty of Health Sciences Human Research Committee. The Boston University Medical Campus IRB (H-41879) reviewed the research and made the determination that it was not human subjects research and therefore had no requirement to obtain consent.

## Results

After excluding participants with implausible values of systolic or diastolic blood pressure, we identified 1,994 incident hypertension cases using the criteria for incident hypertension described below (S1 Fig). Our final sample size for each incident cohort was 639 for wave two, 495 for wave three, 441 for wave four, and 419 for wave five. The proportion of women in each incident cohort decreased over time, from 55.7% in wave two to 44.4% in wave five. There were minor changes in racial composition across the waves, with an overall decrease in the proportion of participants of African race from 80.3% in wave two to 78.3% in wave five. Participants identifying as coloured increased from 15.8% to 18.1% and those of Asian/Indian descent remained stable (1.4% in wave two and 1.9% in wave five) (Table 1).

**Table 1:** Characteristics of NIDS incident hypertension cohorts.

	Cohort 1	Cohort 2	Cohort 3	Cohort 4
	N = 639	N = 495	N = 441	N = 419
Variable	n (%)	n (%)	n (%)	n (%)
Age				
35–44 years	205 (32.1%)	177 (35.8%)	163 (37.0%)	169 (40.3%)
45–59 years	283 (44.3%)	216 (43.6%)	194 (44.0%)	179 (42.7%)
60–74 years	151 (23.6%)	102 (20.6%)	84 (19.0%)	71 (16.9%)
Sex				
Male	210 (32.9%)	205 (41.4%)	164 (37.2%)	189 (45.1%)
Female	429 (67.1%)	290 (58.6%)	277 (62.8%)	230 (54.9%)
Race				
African	513 (80.3%)	412 (83.2%)	335 (76.0%)	328 (78.3%)
Coloured	101 (15.8%)	53 (10.7%)	74 (16.8%)	76 (18.1%)
Asian/Indian	9 (1.4%)	14 (2.8%)	7 (1.6%)	8 (1.9%)
White	16 (2.5%)	16 (3.2%)	25 (5.7%)	7 (1.7%)
Residency				
Rural	342 (53.5%)	268 (54.1%)	218 (49.4%)	200 (47.7%)
Urban	297 (46.5%)	227 (45.9%)	223 (50.6%)	219 (52.3%)
Household Income Quartile				
1 (poorest)	171 (26.8%)	118 (23.8%)	85 (19.3%)	92 (22.0%)
2	208 (32.6%)	146 (29.5%)	121 (27.4%)	103 (24.6%)
3	162 (25.4%)	125 (25.3%)	138 (31.3%)	140 (33.4%)
4 (richest)	98 (15.3%)	106 (21.4%)	97 (22.0%)	84 (20.0%)
Medical Aid Coverage				
No Medical Aid Coverage	581 (90.9%)	429 (86.7%)	382 (86.6%)	373 (89.0%)
Yes Medical Aid Coverage	58 (9.1%)	66 (13.3%)	59 (13.4%)	46 (11.0%)
Education				
No school or up to Grade 9	477 (74.6%)	328 (66.3%)	245 (55.6%)	224 (53.5%)
Schooling above Grade 9	162 (25.4%)	167 (33.7%)	196 (44.4%)	195 (46.5%)

### Trends in the incident hypertension cascade

**Fig 2:**
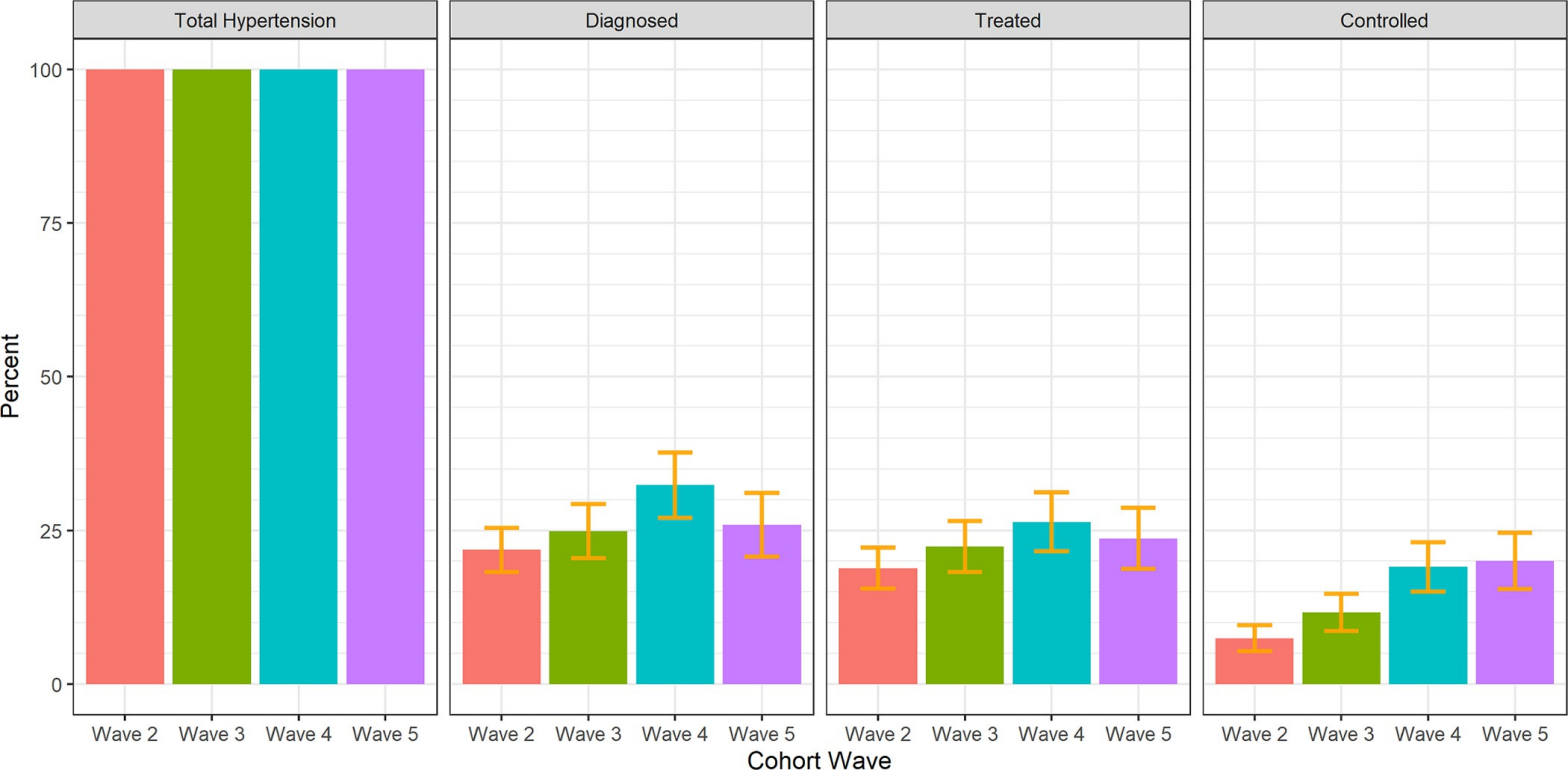
Incident Hypertension Care Cascades, South Africa 2011–2017.

**Fig 3:**
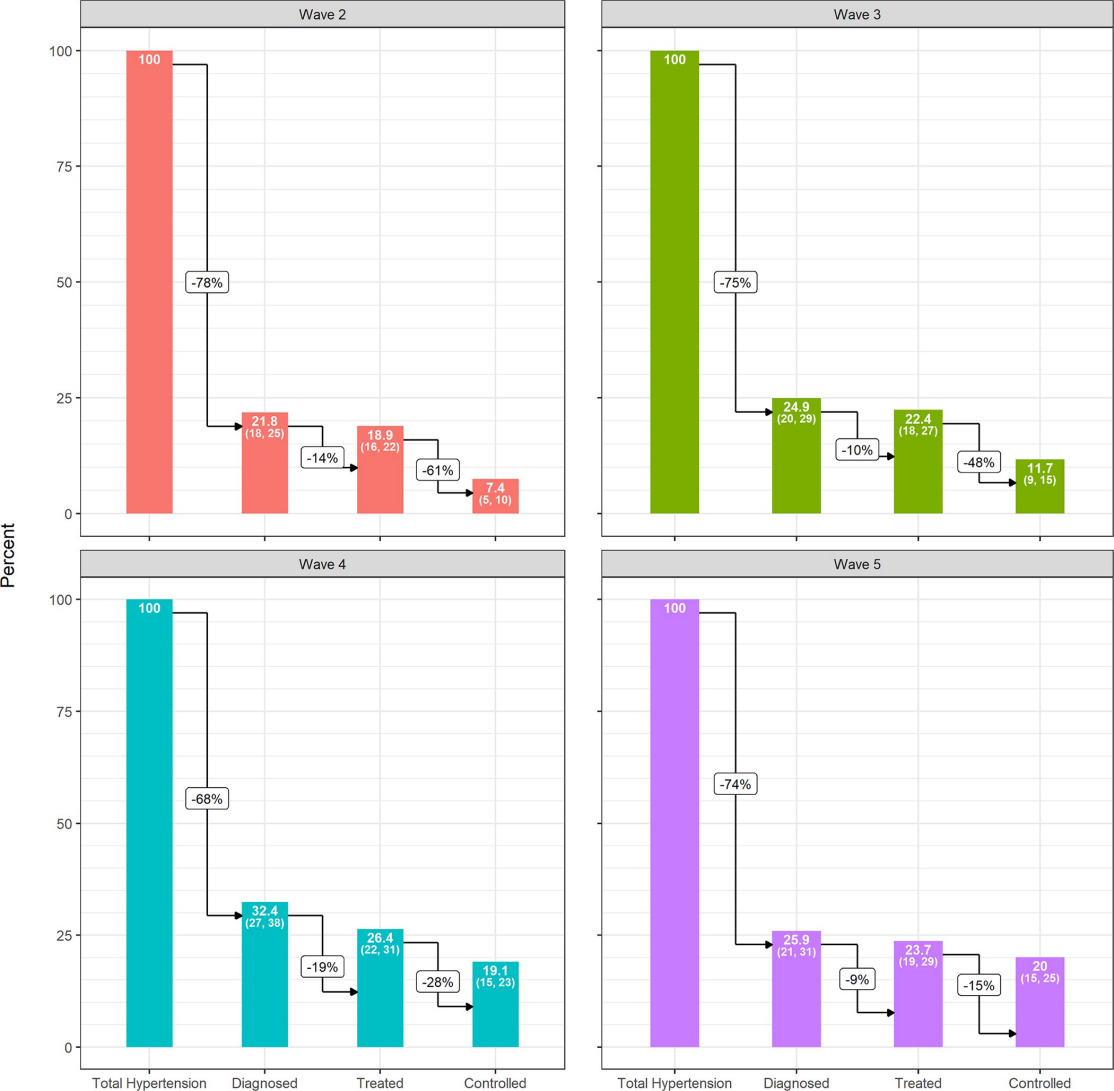
Incident Hypertension Care Cascades by Wave, South Africa 2011–2017.

Fig 2 shows the hypertension care cascades for each of the four incident cohorts. The proportion of those who were hypertensive who were found to be diagnosed increased from 21.8% (95% CI: 18–25%) in wave two, to 24.9%% (95% CI: 20–29%) in wave three and improved again in wave four to 32.4% (95% CI: 27–36%) (Fig 3). It decreased to 25.9% (95% CI: 21–31%) in wave five. The proportion of those who were hypertensive who were diagnosed and treated increased from wave two to five (W2: 18.9%, W3: 22.4%, W4: 26.4%, W5: 23.7%), though there were reductions in the proportion of those who were on treatment from wave four to five. The proportion of the incident hypertensive population who successfully moved through the cascade from diagnosis to hypertension control increased steadily over time, from 7.4% (95% CI: 5–10%) in wave two, 11.7% (95% CI: 9–15%) in wave three, 19.1% (95% CI: 15–23%) in wave four to 20.0% (95% CI: 15–25%) in wave five.

The burden of unmet need for hypertension care was estimated to be 92.6% for the wave two incident cohort. In the wave two cohort, 21.8% of new cases of hypertension reported being diagnosed prior to the study interview. Of those who were diagnosed, 86% reported taking anti-hypertensive medication. Of those on treatment, 39% had a normal blood pressure. This translates to 7.4% of all new hypertension cases in wave two being controlled, and 92.6% uncontrolled. This burden of unmet need, although still great, fell substantially to 80.9 uncontrolled in wave 5.

Among newly hypertensive patients across all four incident cohorts, 74.5% were undiagnosed (Table 2). An estimated 3.4% were diagnosed, 8.5% were treated and 13.5% were controlled. Overall, there were minor changes in the incidence of hypertension from wave two to four, while the percentage of participants who progressed to being controlled increased. We did not observe meaningful differences between different age groups or by race. Women (17.1%) were more likely to have their blood pressure controlled than men (7.9%). When considering income quartiles, those in the highest quartile had the highest proportion of those with controlled hypertension (16.7%). Education above grade 9 was not associated with improved diagnosis or treatment, but had higher rates of control (18.2%) when compared with those who had less education (12.9%). Similarly, medical aid coverage was associated with higher rates of control (18.4%) compared to those with no medical aid coverage (12.9%).

**Table 2:** Hypertension incidence, diagnosis, treatment and control, South African Adults aged 35–74, 2010–2017.

Incident Hypertension					
		Undiagnosed	Diagnosed, Untreated	Treated, Uncontrolled	Treated, Controlled
Variable	Total Incident Cases	%(n)	%(n)	%(n)	%(n)
All					
Crude	1994	74.2% (1480)	3.4% (68)	8.7% (174)	13.6% (272)
Age Adjusted	1994	74.5% (1480)	3.4% (68)	8.5% (174)	13.5% (272)
Survey Year					
2011	639	78.6% (496)	2.9% (19)	11.1% (76)	7.4% (48)
2012	495	74.4% (371)	2.4% (12)	11.2% (54)	12.0% (58)
2014	441	67.4% (295)	6.0% (27)	6.8% (30)	19.8% (89)
2017	419	74.7% (318)	2.4% (10)	3.4% (14)	19.6% (77)
Age					
35–44	714	81.0% (578)	3.4% (24)	4.3% (31)	11.3% (81)
45–59	872	70.8% (620)	3.6% (31)	9.9% (85)	15.7% (136)
60–74	408	68.9% (282)	3.3% (13)	14.1% (58)	13.7% (55)
Sex					
Male	768	83.0% (635)	3.3% (25)	5.8% (47)	7.9% (61)
Female	1226	69.1% (845)	3.5% (43)	10.2% (127)	17.1% (211)
Race					
African	1588	74.2% (1171)	3.6% (57)	8.9% (145)	13.4% (215)
Coloured	304	75.7% (231)	3.6% (11)	8.5% (24)	12.2% (38)
Asian/Indian	38	80.0% (29)	* (*)	8.3% (3)	11.7% (6)
White	64	84.6% (49)	* (*)	1.9% (2)	13.5% (13)
Income quartile					
1 (poorest)	466	78.7% (363)	2.6% (12)	7.7% (39)	11.0% (52)
2	578	73.3% (423)	3.4% (20)	10.3% (61)	13.1% (74)
3	565	73.2% (417)	4.8% (28)	7.4% (42)	14.6% (78)
4 (richest)	385	72.4% (277)	2.0% (8)	8.9% (32)	16.7% (68)
Location					
Rural	1028	75.9% (769)	2.9% (31)	8.9% (98)	12.4% (130)
Urban	966	72.5% (711)	3.8% (37)	8.7% (76)	14.9% (142)
Medical Aid Coverage					
No Medical Aid Coverage	1765	75.0% (1316)	3.5% (61)	8.7% (158)	12.9% (230)
Yes Medical Aid Coverage	229	69.5% (164)	2.9% (7)	9.2% (16)	18.4% (42)
Education					
No school or up to Grade 9	1274	75.0% (934)	3.3% (43)	8.8% (131)	12.9% (166)
Schooling above Grade 9	720	71.8% (546)	3.0% (25)	7.0% (43)	18.2% (106)
Provinces					
Western Cape	231	77.5% (183)	4.1% (9)	5.1% (11)	13.4% (28)
Eastern Cape	295	75.1% (217)	3.9% (13)	12.4% (38)	8.6% (27)
Northern Cape	167	74.0% (123)	4.3% (8)	7.3% (12)	14.3% (24)
Free State	107	66.1% (70)	5.3% (6)	9.2% (10)	19.3% (21)
KwaZulu-Natal	560	74.1% (412)	2.3% (13)	9.4% (55)	14.1% (80)
North West	123	73.3% (93)	2.3% (3)	8.6% (10)	15.8% (17)
Gauteng	212	69.9% (155)	4.5% (9)	9.4% (15)	16.1% (33)
Mpumalanga	123	78.1% (95)	2.9% (3)	5.7% (9)	13.3% (16)
Limpopo	176	74.5% (132)	2.5% (4)	7.7% (14)	15.4% (26)
*No Observations					

Inc = Incidence, SE = Standard Error. Hypertension defined as a blood pressure equal to or above 140mmHg systolic or 90mmHg diastolic.

### Predictors of hypertension cascade progression

Table 3 reports factors associated with having hypertension diagnosed (Table 3). Survey year was not associated with a change in rate of diagnosis. Women were 1.73 (95%CI: 1.42, 2.21) times more likely to be diagnosed than their male counterparts. Increased age was associated with higher rates of diagnosis (incident rate ratio (IRR) 1.54 95% CI: 1.17, 2.02 for those aged 60–74 compared to those aged 35–44). Predictors of being on treatment closely resembled those of being diagnosed. Women were more likely than men to be on treatment (1.89 95% CI: 1.52,2.36) and those aged 60–74 were 1.63 (95% CI: 1.22,2.19) times more likely to receive treatment than their counterparts aged less than 45 years.

**Table 3:** Predictors of incident hypertension control in South Africa, 2011–2017.

	Diagnosis			Treatment			Control		
Variable	IRR	95% CI	Adj%	IRR	95% CI	Adj%	IRR	95% CI	Adj%
Cohort									
Cohort 1 (Wave 2)	1		22%	1		19%	1		7%
Cohort 2 (Wave 3)	1.14	(0.9,1.46)	25%	1.19	(0.92,1.53)	22%	1.57	(1.07,2.31)	12%
Cohort 3 (Wave 4)	1.48	(1.17,1.88)	32%	1.40	(1.08,1.81)	26%	2.56	(1.79,3.66)	19%
Cohort 4 (Wave 5)	1.19	(0.91,1.54)	26%	1.26	(0.95,1.66)	24%	2.69	(1.86,3.9)	20%
Sex									
Male	1		18%	1		14%	1		8%
Female	1.73	(1.42,2.12)	31%	1.89	(1.52,2.36)	27%	2.14	(1.6,2.86)	17%
Race									
African	1		26%	1		22%	1		14%
Coloured	0.96	(0.67,1.38)	25%	1.11	(0.75,1.63)	25%	0.97	(0.59,1.58)	13%
Asian/Indian	0.84	(0.42,1.66)	22%	0.92	(0.47,1.83)	20%	1.06	(0.46,2.46)	14%
White	0.73	(0.4,1.31)	19%	0.94	(0.52,1.7)	21%	1.18	(0.6,2.33)	16%
Age									
35–44 years	1		20%	1		17%	1		12%
45–59 years	1.40	(1.13,1.75)	28%	1.47	(1.16,1.87)	24%	1.23	(0.92,1.65)	15%
60–74 years	1.54	(1.17,2.02)	31%	1.63	(1.22,2.19)	27%	1.11	(0.76,1.64)	13%
Household Income Quartile									
1 (poorest)	1		22%	1		19%	1		12%
2	1.19	(0.93,1.53)	26%	1.18	(0.9,1.55)	23%	1.15	(0.81,1.65)	14%
3	1.23	(0.95,1.59)	27%	1.14	(0.86,1.51)	22%	1.18	(0.82,1.69)	14%
4 (richest)	1.22	(0.9,1.67)	27%	1.30	(0.93,1.8)	25%	1.30	(0.86,1.98)	15%
Residency									
Rural	1		27%	1		23%	1		14%
Urban	1.08	(0.86,1.36)	25%	1.10	(0.86,1.4)	21%	1.10	(0.81,1.5)	13%
Medical Aid Coverage									
No Medical Aid Coverage	1		25%	1		22%	1		13%
Yes Medical Aid Coverage	1.11	(0.81,1.53)	28%	1.10	(0.78,1.56)	24%	1.23	(0.82,1.87)	16%
Known Comorbidities									
No Comorbidities	1		22%	1		19%	1		11%
Yes Comorbidities	2.37	(1.93,2.91)	52%	2.42	(1.94,3.01)	46%	2.66	(2.01,3.51)	30%
Education									
No school or up to Grade 9	1		26%	1		23%	1		14%
Schooling above Grade 9	0.94	(0.75,1.17)	25%	0.91	(0.72,1.16)	21%	0.89	(0.66,1.2)	13%
Provinces									
Western Cape	1		21%	1		16%	1		11%
Eastern Cape	1.24	(0.79,1.92)	26%	1.39	(0.86,2.26)	22%	0.86	(0.46,1.61)	10%
Northern Cape	1.16	(0.76,1.76)	25%	1.20	(0.75,1.91)	19%	1.17	(0.67,2.05)	13%
Free State	1.52	(0.91,2.54)	33%	1.74	(0.99,3.05)	28%	1.67	(0.85,3.28)	19%
KwaZulu-Natal	1.23	(0.79,1.91)	26%	1.54	(0.95,2.5)	25%	1.32	(0.73,2.38)	15%
North West	1.22	(0.72,2.09)	26%	1.53	(0.86,2.74)	24%	1.36	(0.66,2.77)	15%
Gauteng	1.32	(0.83,2.09)	28%	1.49	(0.9,2.47)	24%	1.41	(0.77,2.58)	16%
Mpumalanga	1.06	(0.61,1.82)	23%	1.29	(0.72,2.32)	21%	1.15	(0.56,2.37)	13%
Limpopo	1.12	(0.67,1.87)	24%	1.40	(0.8,2.45)	22%	1.33	(0.67,2.63)	15%
Previous Wave Average Diastolic BP	1.00	(0.99,1.01)		1.00	(0.98,1.01)		0.99	(0.97,1.01)	
Previous Wave Average Systolic BP	1.00	(0.99,1.01)		1.00	(0.99,1.01)		1.00	(0.98,1.01)	

IRR, Incidence Rate Ratio.

Regressions were implemented on the full sample of participants with incident hypertension.

Multiple factors were found to be important in hypertension control (Table 3). Participants who became newly hypertensive between 2014 and 2017 (wave five) had 2.69 (CI: 1.86, 3.9) times more chances of being controlled compared to those who became newly hypertensive between 2008 and 2011 (wave two). Women were more likely than men to have a controlled blood pressure (IRR: 2.14, 95% CI: 1.60, 2.86) in our incident cohorts. White participants (IRR: 1.18, 95% CI: 0.66, 2.33) had higher rates of being controlled than did African participants. Among those with incident hypertension, those aged 45–59 years were more likely to have their blood pressure controlled (IRR 1.23, 95% CI: 0.92, 1.65) than were those aged 35–44 years. Finally, survey year was also found to be associated with a higher predictive probability of having a controlled blood pressure, increasing from 7% (95% CI: 5.3, 9.6) in survey year 2010, to 12% (95% CI: 8.6, 14.7) in 2012, 19% (95% CI: 15.0, 23.1) in 2014 and 20% (95% CI: 15.4, 24.6) in 2017. Having known comorbidities was associated with higher rates of being diagnosed (IRR 2.37, 95% CI: 1.93, 2.91), treated (IRR 2.42, 95% CI: 1.94, 3.01) and controlled (IRR 2.66, 95% CI: 2.01, 3.51). These findings were consistent when we included sample weights (S1 Table).

## Discussion

Using the novel method of incident care cascades, we found that the proportion of people with hypertension who successfully progressed to controlled blood pressure tripled between 2011 and 2017 in South Africa. There remained a very high burden of unmet need, however, with the absolute rate of control—a measure of “met” need—reaching only 20% of those living with hypertension.

Our finding of the greatest losses in the incident hypertension care cascades coming before diagnosis is consistent with other literature from sub-Saharan Africa, which reports that the biggest unmet burden of need lies between developing disease and diagnosis [8,26–29]. This burden is expected to grow [30]. Other literature also shows that South Africa faces a substantially higher burden of hypertension than other sub-Saharan countries, but that South Africans with hypertension may be more likely to be aware of their condition [31]. Using data from the South African National Health and Nutrition Survey (SANHANES 2011–2012), Berry et al. found that 71.8% of South Africans with hypertension did not reach the diagnosis phase of the cascade, while Ware et al found that 58% of hypertensive South Africans were unaware of their diagnosis [28,32]. There are many reasons for this loss in South Africa and other low-middle income settings, such as the fact that individuals rarely seek health care for asymptomatic conditions like hypertension and that men in particular show poor health seeking behavior [7]. The SANHANES found that the mean duration since health care was last received among South Africans to be between 1.6 and 2.2 years, with 20.7% of women and 27.5% of men reporting never have sought access to public health care [33]. These differences in health seeking behavior may at least in part explain the different odds of having a controlled blood pressure between men and women as observed in our data.

Encouragingly, we found that control of hypertension in our incident cascades improved over time, from 7.4% in wave two to 20.0% in wave five. These changes were, however, not uniform, with those in the third and fourth income quartile being 1.18 and 1.30 times more likely to successfully move through the cascade than those in lower income quartiles and those with education above grade 9 being 2.66 times more likely than those with less education. One reason for cascade improvements might be growing efforts in South Africa to leverage the robust HIV infrastructure to provide primary care for other conditions [34,35]. Primary care programs for HIV have been shown to improve NCD markers for quality care in multiple settings across sub-Saharan Africa, with an undetectable viral load associated with a lower systolic blood pressure and patients on ART having improved rates of progression through various phases of NCD cascades [36,37]. This may be especially important in South Africa, which faces a high burden of HIV [38]. With the adoption of universal HIV treatment in South Africa in 2016, there have been substantial increases in HIV treatment uptake and viral load suppression, suggesting that healthcare infrastructure has been able to scale up care delivery that can also be directed towards diseases like hypertension [39]. We should note, however, that improvements in the HIV cascade do not necessarily translate to better NCD care. Muddu et al. found that in Uganda, while 90.3% of HIV patients progressed to viral suppression, only 24.3% of the same patient population had a controlled blood pressure [26]. More explicit intervention may be needed to utilize HIV infrastructure to routinely screen people for hypertension. Encouragingly, countries like Malawi and Kenya have enacted also policies that allow for integration of HIV and NCD care. Importantly, some of these interventions target lifestyle changes for prevention of NCDs for the entire population, not only people living with HIV [40]. Modelling studies conducted in Kenya suggest that while the cost of health system changes to accommodate integrated care is high, it can be cost effective in most scenarios, while preventing 116,600 cardiovascular events and 43,600 cardiovacular related deaths by 2033 if 50% of those diagnosed through integration programs were to receive treatment [41].

In addition to leveraging HIV-related infrastructure, hypertension control may also be improving due to NCD-focused initiatives by the South African National Department of Health (NDoH) [11]. These include the adoption of the integrated chronic disease management (ICDM) model, that included facility reorganization, outreach teams for assisted self-management, and health promotion [35]. Evaluation of the 2011 ICDM pilot through a controlled interrupted time-series analysis revealed modest gains, with only a 7.0% greater probability of having controlled blood pressure among patients in ICDM facilities, compared to routine-care facilities, possibly due to poor referrals, poor defaulter tracing, and problems with prepacking of medications, clinic appointments and patient waiting times [12,35]. The Ideal Clinic Programme, launched in 2013, aimed to address some of these concerns by establishing standards for infrastructure, adequate staff, adequate medicine and supplies, and administrative processes [11,16]. The South African government has also adopted policy level changes with the *Strategic Plan for the Prevention and Control of Non-Communicable Diseases 2013–2017* [10]. Results of this strategy included legislation that limited excessive salt and sugar intake, safe green spaces with exercise equipment to encourage physical activity and national guidelines on the management of hypertension that advocate strongly for lifestyle modification as a first line of treatment [10,24,42,43]. Some of these macro-level changes may have led to improved control of hypertension over time, as our analysis found. Whatever the causes, the WHO NCD Country profile for South Africa for 2017 shows that there have been substantial strides towards providing continuous access to anti-hypertensive medications at primary health care facilities [44]. Our results both confirm the value of the policy and practice revisions and underscore the large challenge that remains.

It is possible that some of the progress made over recent years was lost due to disruptions in routine care during the COVID-19 pandemic [45]. According to a WHO survey completed by 155 countries in May 2020, more than half of the countries surveyed reported disruptions in service delivery for hypertension, mainly due to staff being reassigned to support expanding health system efforts to treat COVID-19 [46]. Other common reasons for disruptions were decreased availability of public transportation and the cancellation of treatments planned prior to the pandemic [46]. The long-term effects on the hypertension care cascade in South Africa remain to be seen.

Our study had several limitations. Diagnosis and treatment were self-reported, and patients on medications for hypertension may be more likely to report diagnosis than those not on medications. We were not able to account for lifestyle modification in our treatment variable or assess the outcomes for people who were excluded due to missing variables. Survey effects would be of concern in a prevalent cascade, as interviewers would direct patients to care if their blood pressure were found to elevated as part of the study, thereby possibly influencing participant’ health seeking behaviour and how they progress through the cascade. Our incident cascade mitigates this problem by only considering new hypertensive cases. Finally, our study may be subject to selection biases in the pool of eligible participants over waves. However, this issue is at least partially mitigated by our adjustments for differences in baseline health status across incident cohorts in the multivariable regression analyses. Another potential mitigating factor is that the NIDS sampled from the same households across waves, which may enhance comparability of the incident cohorts, as household members would to an extent have similar risk factors associated with hypertension. Finally, the relatively small sample size of this analysis may limit generalizability. Therefore, the findings of this study should be confirmed in other national cohorts.

## Conclusion

In conclusion, the novel use of incident cohorts in this study allowed the preservation of longitudinal data for analysis, thereby allowing us to give a more accurate depiction of current system performance by negating the effects of longstanding prevalent cases. We found that the proportion of people with controlled hypertension increased over time, but that there still exists a large burden of unmet need for diagnosis, treatment, and control. Improvement in controlled hypertension over the study time period might be due to the South African National Department of Health moving towards a more integrated approach in the management of HIV and NCDs through the Ideal Clinic program. Identifying key leverage points within the Ideal Clinic program may thus offer a promising avenue for further investigation to inform policy changes in similar settings.

## Supporting information

S1 Fig Flow diagram of exclusion criteria. (TIF)

S2 Fig Diagram illustrating how incident cohorts were created. (TIF)

S1 Table Predictors of incident hypertension control in South Africa, 2011–2017, weighted. (RTF)
