# Supplementary figures and images for: Health systems performance for hypertension control using a cascade of care approach in South Africa, 2011–2017
Source: PLOS Glob Public Health. 2023 Sep 7;3(9):e0002055. doi: 10.1371/journal.pgph.0002055 (PMC10484448; doi:10.1371/journal.pgph.0002055)

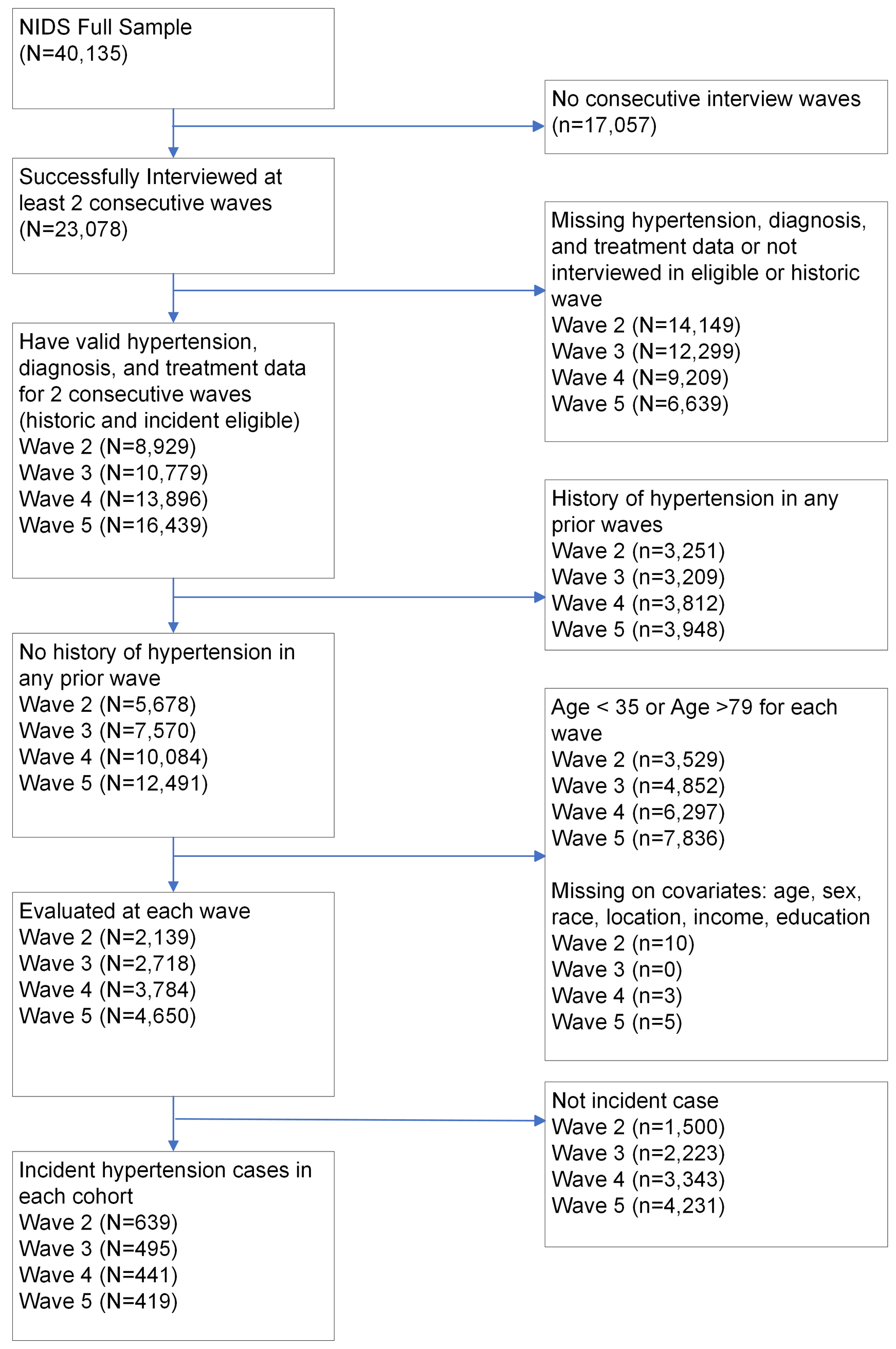

Supplement: S1 Fig — (TIF) [file pgph.0002055.s001.tif]

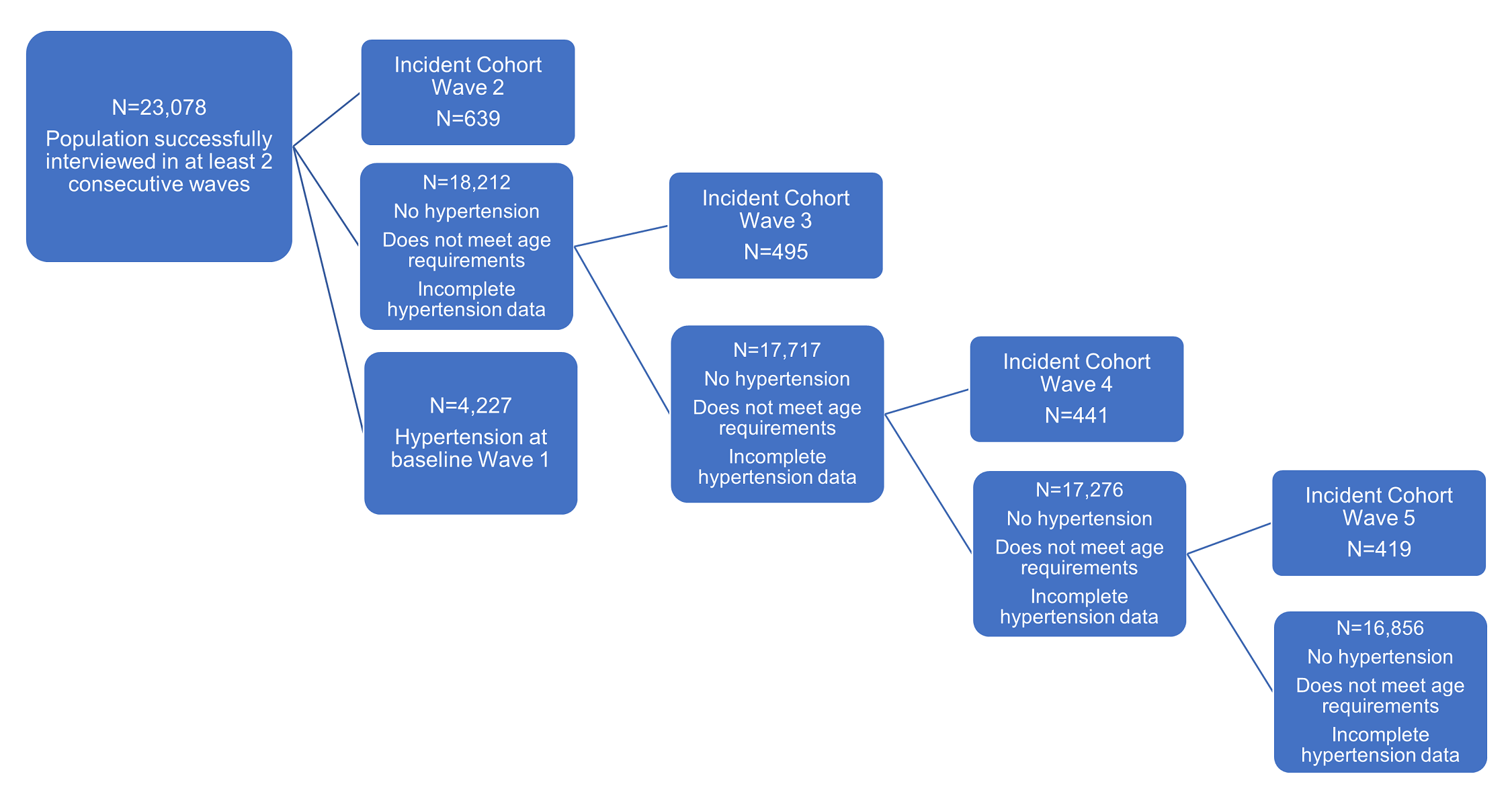

Supplement: S2 Fig — (TIF) [file pgph.0002055.s002.tif]
