# Supplementary material for: Health systems performance for hypertension control using a cascade of care approach in South Africa, 2011–2017
Source: PLOS Glob Public Health. 2023 Sep 7;3(9):e0002055. doi: 10.1371/journal.pgph.0002055 (PMC10484448; doi:10.1371/journal.pgph.0002055)
Supplement: S1 Table — (RTF) [file pgph.0002055.s003.rtf]

	Diagnosis	Treatment	Control	
Variable	IRR	95% CI	Adj%	IRR	95% CI	Adj%	IRR	95% CI	Adj%	
Cohort	
Cohort 1 (Wave 2)	1		19%	1		16%	1		7%	
Cohort 2 (Wave 3)	1.12	(0.82,1.52)	22%	1.27	(0.92,1.76)	21%	1.72	(1.05,2.8)	13%	
Cohort 3 (Wave 4)	1.39	(1.03,1.87)	27%	1.36	(0.98,1.89)	22%	2.23	(1.44,3.48)	16%	
Cohort 4 (Wave 5)	1.06	(0.73,1.54)	21%	1.12	(0.75,1.66)	18%	2.26	(1.36,3.76)	17%	
Sex	
Male	1		13%	1		10%	1		6%	
Female	2.30	(1.73,3.05)	29%	2.52	(1.85,3.42)	26%	2.98	(1.99,4.45)	17%	
Race	
African	1		23%	1		19%	1		12%	
Coloured	0.89	(0.55,1.43)	20%	0.99	(0.57,1.7)	19%	0.66	(0.34,1.27)	8%	
Asian/Indian	0.52	(0.18,1.49)	12%	0.56	(0.19,1.68)	11%	0.87	(0.28,2.71)	10%	
White	0.95	(0.48,1.87)	21%	1.11	(0.55,2.23)	21%	1.53	(0.72,3.24)	18%	
Age	
35-44 years	1		18%	1		15%	1		12%	
45-59 years	1.29	(0.94,1.77)	24%	1.39	(0.99,1.95)	21%	1.17	(0.77,1.77)	14%	
60-74 years	1.33	(0.9,1.98)	25%	1.46	(0.95,2.25)	22%	0.71	(0.41,1.23)	8%	
Household Income Quartile	
1 (poorest)	1		18%	1		16%	1		11%	
2	1.31	(0.92,1.85)	23%	1.23	(0.83,1.8)	20%	1.03	(0.61,1.73)	11%	
3	1.26	(0.87,1.83)	23%	1.21	(0.8,1.82)	20%	0.95	(0.54,1.67)	10%	
4 (richest)	1.25	(0.8,1.96)	22%	1.22	(0.78,1.91)	20%	1.37	(0.8,2.35)	15%	
Residency	
Rural	1		21%	1		18%	1		12%	
Urban	0.93	(0.67,1.3)	23%	0.88	(0.6,1.28)	21%	0.98	(0.6,1.59)	13%	
Medical Aid Coverage	
No Medical Aid Coverage	1		21%	1		18%	1		12%	
Yes Medical Aid Coverage	1.14	(0.76,1.72)	24%	1.20	(0.78,1.85)	22%	1.24	(0.77,2)	14%	
Known Comorbidities	
No Comorbidities	1		18%	1		16%	1		9%	
Yes Comorbidities	2.88	(2.31,3.58)	52%	3.04	(2.35,3.93)	47%	3.78	(2.78,5.13)	36%	
Education	
No school or up to Grade 9	1		23%	1		20%	1		14%	
Schooling above Grade 9	0.92	(0.67,1.26)	21%	0.96	(0.7,1.31)	19%	0.78	(0.51,1.19)	11%	
Provinces	
Western Cape	1		16%	1		13%	1		7%	
Eastern Cape	1.31	(0.75,2.32)	21%	1.33	(0.72,2.47)	18%	0.98	(0.45,2.15)	7%	
Northern Cape	1.45	(0.82,2.55)	23%	1.41	(0.74,2.69)	19%	1.98	(0.85,4.61)	14%	
Free State	1.64	(0.9,2.97)	26%	1.90	(1,3.61)	26%	2.93	(1.43,6)	21%	
KwaZulu-Natal	1.29	(0.71,2.32)	21%	1.40	(0.72,2.71)	19%	1.87	(0.84,4.18)	13%	
North West	1.42	(0.72,2.79)	23%	1.65	(0.79,3.43)	22%	1.74	(0.69,4.41)	12%	
Gauteng	1.75	(1.04,2.94)	28%	1.74	(0.97,3.12)	23%	2.61	(1.36,4.98)	18%	
Mpumalanga	1.22	(0.64,2.3)	20%	1.34	(0.67,2.68)	18%	1.83	(0.84,3.95)	13%	
Limpopo	1.15	(0.6,2.19)	18%	1.30	(0.64,2.62)	17%	2.06	(0.86,4.9)	14%	
	
Previous Wave Average Diastolic BP	0.99	(0.98,1.01)		0.99	(0.97,1.01)		0.98	(0.96,1.01)		
Previous Wave Average Systolic BP	1.00	(0.99,1.02)		1.00	(0.98,1.01)		1.00	(0.99,1.02)		
